# The relationship between cognitive decline and a genetic predictor of educational attainment

**DOI:** 10.1016/j.socscimed.2019.112549

**Published:** 2019-10

**Authors:** Xuejie Ding, Nicola Barban, Felix C. Tropf, Melinda C. Mills

**Affiliations:** a Department of Sociology, University of Oxford, UK; b Nuffield College, University of Oxford, UK; c Institute for Social and Economic Research (ISER), University of Essex, UK; d Center for Research in economics an Statistics (CREST), École Nationale de la Statistique et de L'administration Économique (ENSAE), France; e Leverhulme Centre for Demographic Science, University of Oxford, UK

**Keywords:** Cognitive decline, Educational attainment, Fluid/crystallised intelligence, Genetic predictor, Growth curve modelling, Polygenic risk score

## Abstract

Genetic and environmental factors both make substantial contributions to the heterogeneity in individuals’ levels of cognitive ability. Many studies have examined the relationship between educational attainment and cognitive performance and its rate of change. Yet there remains a gap in knowledge regarding whether the effect of genetic predictors on individual differences in cognition becomes more or less prominent over the life course. In this analysis of over 5000 older adults from the Health and Retirement Study (HRS) in the U.S., we measured the change in performance on global cognition, episodic memory, attention & concentration, and mental status over 14 years. Growth curve models are used to evaluate the association between a polygenic risk score for education (education PGS) and cognitive change. Using the most recent education PGS, we find that individuals with higher scores perform better across all measures of cognition in later life. Education PGS is associated with a faster decline in episodic memory in old age. The relationships are robust even after controlling for phenotypic educational attainment, and are unlikely to be driven by mortality bias. Future research should consider genetic effects when examining non-genetic factors in cognitive decline. Our findings represent a need to understand the mechanisms between genetic endowment of educational attainment and cognitive decline from a biological angle.

## Highlights

• Older adults with higher scores perform better across all measures of cognition.
• The relationship is robust after controlling for phenotypic educational attainment.
• The genetic effect on episodic memory diminishes with age.
• Future research should consider genetic effects when examining cognitive decline.

Cognitive competencies tend to decline with age. Interpersonal variability in age-related cognitive decline is not fully understood: while some people experience substantial deterioration in cognitive function, others maintain better cognitive status despite the presence of considerable brain deterioration (Stern, 2009). Cognitive decline threatens independence and quality of life for older adults (Williams and Kemper, 2010). With an ageing population, both in the U.S. and worldwide, cognitive decline is an emerging health and social issue, especially since older individuals are increasingly taking additional responsibility for financial and medical decisions. Understanding the predictors that contribute to the variation in the trajectories of cognitive ageing has important biological and public health implications. It may not only provide insight into the deterioration of cognitive function, but also enable us to identify individuals at high risk of rapid decline and the development of personalised strategies for prevention of cognitive-skill related comorbidities.

Genetic, socioeconomic and behavioural risk factors all make substantial contributions to the heterogeneity in individuals’ level of cognitive ability. Twin and family-based studies indicate that at least moderate proportion of the differences in most domains of cognitive ability is associated with genetic factors (Bouchard and McGue, 2003; Rietveld et al., 2014). Social scientists have shown that the relationship between education and cognition is in part due to the causal effect of schooling. This relationship can also be due to genetic confounding. Recent genome-wide association studies (GWAS) found that the genetic components of general cognitive functions are about 20–30% heritable (Davies et al., 2016). Higher educational attainment may allow individuals to cope more effectively with age-related brain deterioration, and thus perform better on cognitive tasks in later life (Lenehan et al., 2015; Rietveld et al., 2014; Scarmeas and Stern, 2004). Recent GWAS have discovered molecular genetic associations with education (Lee et al., 2018) and general cognition (Davies et al., 2018). The polygenic score of education constructed by Lee et al. (2018) explains 11–13% of the variance of educational attainment and 7–10% of the variance of cognitive performance, suggesting that the phenotypes have shared genetic basis (Marioni et al., 2014; Okbay et al., 2016; Rietveld et al., 2014). These findings suggest that common genetic effects may account for some of the observed association between education and cognitive ability.

Genetic variants that promote educational attainment – those that influence brain development and neuron-to-neuron communication, for example – may have an effect on cognitive functioning throughout the life course (Lee et al., 2018). The magnitude of their effects may also change with age. For example, previous research attempting to examine the importance of genetic risks across the life course has shown that ageing magnifies genetic effects on cognitive ability (Laukka et al., 2013; Li et al., 2013; Papenberg et al., 2014; Papenberg et al., 2015). The rationale is that the association between brain resources and cognitive ability is nonlinear, and that genetic variation is more influential on performance differences during normal ageing when the brain starts to lose neurochemical and structural resources (Lindenberger et al., 2008). However, the majority of previous studies suffers from shortcomings regarding research design and methodology, including cross-sectional data sources (Li et al., 2013; Nagel, 2008), a small number of assessed genetic variants (Bretsky et al., 2003; Schiepers et al., 2012), and focus on a narrow period of the life course (Moorman et al., 2018). Therefore, it remains largely unknown how affect the trajectory of cognitive abilities and its rate of change among older adults.

The objective of this study is thus to investigate whether the polygenic score for education are associated with later life cognitive functions and cognitive decline independently among middle-aged and older adults in the United States. We measure cognition and its decline both separately in the domains of episodic memory, attention and calculation, and mental status, and as an index measuring general cognition. We use polygenic scores constructed for the Health and Retirement Study (HRS) that summarise an individual's cumulative genetic predictor to educational attainment. The polygenic scores for educational attainment (hereafter, education PGS) are constructed by adding the effect-size-weighted risk alleles across the genome associated with education based on the third and most recent educational attainment GWAS consortium paper by Lee et al. (2018), which used data from 1.1 million participants and identified 1271 lead genetic variants. The education PGS correlates with years of education (β = 0.8; se = 0.03) with a predictive power of 10% in our HRS sample (see supplementary material for more details). This research tackles the following three research questions: 1) How are the education PGS associated with level of cognitive function? 2) How does the effect of education PGS on individual differences in cognition change with age? 3) Does the relationship between genes, age, and cognition still hold after controlling for other socioeconomic, behavioural and health factors? We use growth curve analysis across the waves (1998–2014) of the HRS to gain a better understanding of how genes and education operate across the life course as people age.

## 1 Genetics predictors of educational attainment and cognitive decline

Cognitive ability varies among individuals across the life-span. Moreover, the within-person sub-dimensions of cognitive decline at different rates: verbal, numerical and knowledge-based abilities remain relatively stable in late life, while other mental abilities such as memory and processing speed start to deteriorate from middle age or even earlier and at a faster rate (Mustafa et al., 2012; Nisbett et al., 2012). Episodic memory – the ability to encode and retrieve personally experienced events that occurred at a specific place and time (Gabrieli, 1998) – is a type of fluid intelligence that involves the ability to think and reason abstractly. Evidence suggests that episodic memory is independent of pre-existing knowledge, learning and education, and is relatively more sensitive to genetic variability (Smith et al., 2018), for example, the Apolipoprotein E (APOE). On the other hand, mental status, attention and calculation are types of crystallised intelligence, which is formed through accumulating knowledge and experience. As people age and gain new knowledge and understanding, crystallised intelligence tends to increase first and decline more slowly (Salthouse, 2012). As a channel to gain knowledge and skills, education is expected to have a substantial effect on crystallised abilities.

**Fig. 1:**
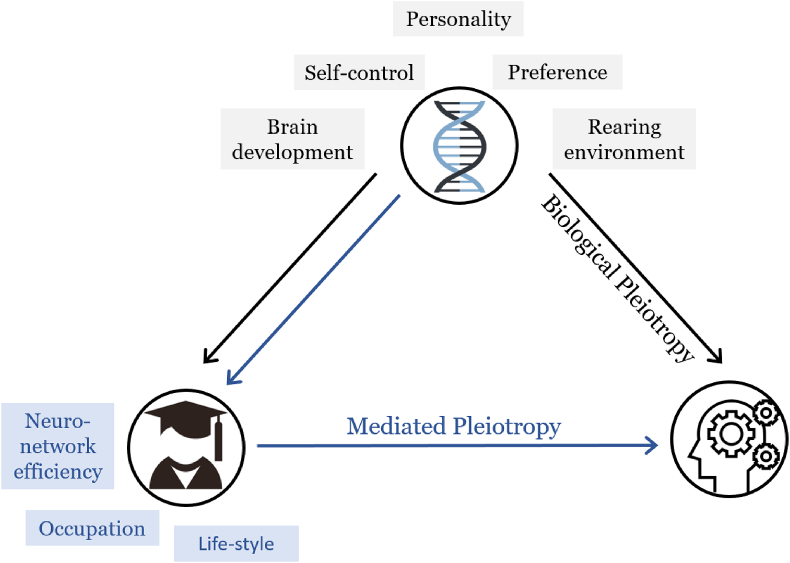
Pleiotropy types and mechanisms between gene, education and cognition.

Wedow et al. (2018) described two pathways (Fig. 1) through which genes could influence social and health outcomes. One pathway for the connection between education PGS and cognitive functions is *biological pleiotropy* (Pickrell et al., 2016). That is, genes contribute to both educational attainment and cognition independently due to underlying biological and latent genetic mechanisms. For example, a person's genetics may influence brain development to affect non-cognitive self-control, interpersonal skills, preferences and behaviours leading to differences in both educational attainment and cognition (Belsky et al., 2016; A. Okbay et al., 2016). In addition, a person's education-increasing genotypes may be associated with parental education-increasing genotypes, which can in turn select the individuals into socially advantaged families that promote both cognitive development and educational attainment (Belsky et al., 2016, 2018; Kong et al., 2018; Lee et al., 2018). This explanation raises the issue that any observed association between educational attainment and cognition may be spurious due to the omitted genetic variable bias.

The second pathway for the association between genetic predictors for education and cognitive status is *mediated pleiotropy.* Well-established evidence suggests that educational level in early life affects the level of cognitive performance in later life (Gatz et al., 2001; Tucker-Drob et al., 2009; Van Dijk, Van Gerven, Van Boxtel, Van der Elst and Jolles, 2008; Zahodne et al., 2011). The potential mechanisms are improved cultural competence and reasoning skills, a more effective use of brain function and cognitive processing, and a healthier occupational environment and lifestyle (Andel et al., 2006; Chen et al., 1999; Kramer et al., 2004). According to this explanation, genes that are causally associated with education affect cognitive performance through the mediated path.

The first research question is therefore whether there is an association between education PGS and the level of cognitive ability? Evaluating this relationship could aid our understanding of the association between educational attainment and cognitive status – to what extent cognition is influenced by educational attainment via biological mechanisms and unobservable confounders related to environmental factors. Drawing from recent advances in GWAS and the theoretical relationship between education and cognitive performance, we hypothesise that education PGS is positively related to the level of cognitive ability in old age, independent of the phenotypic educational attainment (Hypothesis 1).

The relationship between genetics and cognitive rate of change is less straightforward and more domain-specific. Since crystallised intelligence is more dependent on education, we speculate that genetics are more likely to affect attention and concentration, and mental status via mediated pleiotropy. Evidence on whether educational level influences the trajectory of age-related cognitive decline is inconsistent. These inconsistent findings may be due to methodological differences, such as sample characteristics, analytic strategies, type of cognitive measures and decline, or selection and confounding (Foverskov et al., 2018; Gottesman et al., 2014).

Some earlier studies linking education with cognitive change in old age find that lower levels of education are associated with a faster decline in verbal fluency, mental status and general cognition (e.g. Albert et al., 1995; Jacqmin-Gadda et al., 1997; Lyketsos et al., 1999). These studies posit that individuals with a higher level of education use brain networks or cognitive paradigms more efficiently or flexibly, and would exhibit a smaller decline in cognitive function relative to those with a lower level of education (Salthouse et al., 2003). More recent studies cast doubts on whether rates of cognitive decline vary by education in later life. Many suggest that higher levels of education do not attenuate the rate of decline in episodic memory, working memory, processing speed and verbal fluency (Glymour et al., 2012; Gottesman et al., 2014; Karlamangla et al., 2009; Zahodne et al., 2011). Others report that higher education is associated with faster cognitive decline in attention & concentration. (Gottesman et al., 2014; Zahodne et al., 2015). A potential explanation for the lack of positive association between education and rate of cognitive decline is that education raises baseline cognitive performance, which increases the time needed to decline to the pathological threshold. People with higher level of education thus decline at a similar rate to their lower-educated counterparts, or even a faster rate if they rely on specific cognitive domains to compensate for declines in other cognitive domains. In summary, recent evidence shows no association on the phenotypic educational attainment and cognitive decline. Since declines in crystallised domains are less sensitive to the educational protective effect, we extend the phenotypic perspective to genetic inquiry and hypothesise that education PGS are not associated with the rate of cognitive change in crystallised domains (Hypothesis 2).

From a biological perspective, studies demonstrate magnified genetic influence on different types of cognition and the rate of cognitive decline during normal ageing (Tucker-Drob et al., 2014). Meta-analysis suggests increased heritability for episodic memory, working memory and spatial ability from early to late adulthood (Reynolds and Finkel, 2015). The *resource-modulation hypothesis* proposed by Lindenberger et al. (2008) hypothesises that losses of structural and neurochemical brain resources in non-pathological ageing moderate the effects of common genetic variations on cognitive performance. The hypothesis assumes a non-linear function linking brain resources to cognitive abilities, and differences in genetic predictor exert magnifying effects on cognitive functions as brain resources reduce from high to medium levels. Given that episodic memory may be closer to the molecular effects of a gene than cognitive reserve such as education, the rate of change in episodic memory is expected to be more sensitive to genetic effects (Papenberg et al., 2015; Rasch et al., 2010). Older adults, therefore, may benefit more from positive genetic endowment relative to their younger counterparts. Therefore, we hypothesise that a higher level of education PGS may be associated with a lower rate of decline in episodic memory (Hypothesis 3).

Previous research has found evidence that supports the resource-modulation hypothesis. For example, APOE polymorphism is involved in lipid homeostasis and injury repair in the brain (Papenberg et al., 2015): carrying the ε4 allele is a strong risk factor for accelerated cognitive decline in ageing (Filippini et al., 2011; Liu et al., 2010; Zhang and Pierce, 2014). However, the literature to date suffers from a few limitations. First, the majority of past research focuses only on one or a handful of genetic variants such as the aforementioned APOE (Bretsky et al., 2003; Schiepers et al., 2012), brain-derived neurotrophic factor (Ghisletta et al., 2014), catechol-O-methyltransferas (Papenberg et al., 2013) and kidney- and brain-expressed protein (Muse et al., 2014). These studies are controversial as they tend to produce results that are rarely replicable due to their lack of power to detect plausible effects (Benjamin et al., 2012; Chabris et al., 2012). Second, a large number of studies adopt a cross-sectional design (Li et al., 2013; Nagel, 2008); longitudinal studies are rare but necessary to confirm the patterns observed in the cross-sectional data (Papenberg et al., 2015). Third, recent studies using the polygenic scores from GWAS studies and longitudinal data sets tend to focus on cognitive development in young age (Moorman et al., 2018), and at a narrow period of the life course (Ritchie et al., 2019).

In summary, our research studies how genetic variants influence trajectories of cognitive performance across the later lifespan. We overcome the aforementioned limitations by measuring genetic predictors for education using the polygenic score method among over 7000 individuals aged 50 and above and tracked over 16 years.

## 2 Data and methods

### 2.1 Data

The Health and Retirement Study (HRS) began in 1992 and is a biennial, longitudinal survey of a nationally-representative sample of individuals and their spouses aged 50 and above. In 2006 and 2008, the HRS collected genetic (saliva) samples from approximately 84% of participants undergoing face-to-face interviews (12,507 individuals). These DNA samples were genotyped for about 2 million SNPs. This study exploits the longitudinal nature of the HRS to explore cognitive performance trajectories among older adults in the U.S. We use eight waves of HRS data (from 1998 to 2012). Pre-processed datasets included the user-friendly RAND HRS data files (version P) and 1998–2012 HRS Core Files.

### 2.2 Sample

During the period 1998–2012, 8652 respondents were genotyped. Growth curve models typically require at least three waves of repeated measures for each individual (Curran et al., 2010), 2699 (31.2%) respondents whose cognitive performance was measured fewer than three times were removed. Since this study only focuses on age-related cognitive decline, we have differentiated normal cognitive functioning from impaired functioning. A composite score measuring memory and mental status have been constructed (ranging from 0 to 27). Respondents (n = 36) with a score of less than seven exhibited signs of dementia (Crimmins et al., 2011) and were removed. Finally, for the main analysis, only individuals from European and non-Hispanic backgrounds were included. The 5859 remaining respondents had at least three cognitive interviews: 25% had four or fewer interviews, 50% had six or more interviews, providing 34,184 person-wave observations.

### 2.3 Dependent variables – cognitive measures

In the HRS, assessment of cognitive function is based on a reduced version of the telephone interview for the assessment of cognitive status (Desmond et al., 1994), which was derived from the Mini-Mental State Exam (MMSE) (Folstein et al., 1975). The assessment has been validated for use as a screening instrument for cognitive performance. The same cognitive tests were administered during all the included waves of data collection and were used to construct cognitive trajectories for individuals on each test (Herzog and Wallace, 1997).

Episodic memory (EM) was measured by immediate and delayed word recall. Respondents were read a list of ten common words (e.g. hotel, sky, water) and were then asked to recall as many of them as possible both immediately after the list was read and also several minutes later. The score records the total number of words the respondent correctly recalled at each instance and ranges from 0 to 20.

Attention & calculation (A&C) was assessed with the serial 7s subtraction test. The respondents were asked to subtract 7 from 100 and continue subtracting 7 from each subsequent number for a total of five trials. The scores record the correct number of trials (ranging from 0 to 5). The serial 7s subtraction test assessed mixed abilities of attention, calculation and working memory that maintains and manipulates information using short-term memory.

Mental status (MS) was assessed by naming the date, month, year and day of the week (ranging from 0 to 4), backwards counting from 20 (0–2), object naming (0–2), and naming the current president and vice president of the U.S (0–2).

Global cognition (GC) is a summary measure of the cognitive domains mentioned above (ranging from 0 to 35). To provide comparability across all measurements, we rescaled individual and global cognitive variables into a corrected percentage score – based on division by the maximum score and multiplication by 100.

The HRS includes other additional cognitive measures such as Wechsler Adult Intelligence Scale similarities, numeracy, quantitative reasoning and verbal fluency modules. We chose not to include them in our analyses as they were either asked of a small group of respondents or only added to the survey waves recently (Fisher et al., 2017).

### 2.4 Independent variable – education PGS

The education PGS is based on the most recent GWAS results excluding the HRS samples (Lee et al., 2018), from which SNP effects on years of education are obtained. Higher scores predict higher years of education and serve as indicators for a genetic predictor to educational attainment. The education PGS was standardised for the full sample so that effects can be interpreted as a ±1 SD change relative to the sample. The relationship between education PGS, years of education and cognitive functions are presented in Supplementary Material Table S1.

The research method using genetic data may suffer from potential selection bias, as respondents had to live until the 2006–2008 genotyping period. Of the original 37,495 respondents, 28,136 (75%) lived until at least 2006. Death of HRS participants prior to genotype collection in 2006, 2008 and 2010 may cause mortality selection bias. If individuals with lower level of education PGS and worse cognition were more likely to die, the association we estimated on the sample could be confounded (Domingue et al., 2017). To alleviate the concern, we applied the inverse probability weighting to account for mortality selection in our main analyses.

### 2.5 Covariates

Educational attainment is measured in years of education. We control for gender and population stratification for all analyses, as the frequencies of certain genetic variants vary by ancestral background. Ignoring genetic variation due to ancestry may result in population stratification bias when genetic effects are confounded by ancestry. Standard practice in accounting for population stratification using GWAS data is to include as covariates the first few principal components that capture most of the genetic variation due to ancestry. We adjusted for population stratification using the first ten principal components (Price et al., 2006).

### 2.6 Analytical strategy

Growth curve models were used to examine the individual cognitive trajectories of the respondents, which enabled us to study the effect of genetic predictor to educational attainment on the level of cognitive ability and its rate of change. We fit a linear, age-related decline random effect model and allow the age intercept and slope in the models to co-vary. Separate growth curve models were estimated with each cognitive measure as a dependent variable. Random effects included intercept and linear age, with the conventional unstructured covariance. A general specification of the model is

$$Cognition_{ij}=\beta_{0}+\beta_{1}\times \;\left(Age_{j}-\bar{Age}\right)+\;\beta_{2}\times PGS_{i}+\beta_{3}\times PGS_{i}\times \left(Age_{j}-\bar{Age}\right)\;+\beta_{4}*{Χ}_{ij}+\;\beta_{5}*{Χ}_{ij}*\left(Age_{j}-\bar{Age}\right)\;+\;\mu_{ij}+\;\;\mu_{ij}*\;\left(Age_{j}-\bar{Age}\right)+\varepsilon_{\iota }$$

where age is centred around the grand mean (75), $Cognition_{ij}$ represents the cognitive score for person i at age j $,\;\beta_{0}$ is the population mean of cognitive ability at the grand-mean age, $\beta_{1}$ represents the linear fixed effect of age, $\beta_{2}$ represents the effect of education PGS on the cognitive ability, $\beta_{3}$ is the linear effect of education PGS on the change rate of cognitive skills, $\beta_{4}$ and $\beta_{5}$ are the effects of X – a vector including individual covariates – on initial cognitive ability and the growth rate of change. $\sigma_{1}$ and $\sigma_{2}$ are the random intercept and slope. $\mu_{ij}$ and $\mu_{ij}$ are intercept and age variance.

## 3 Results

**Fig. 2:**
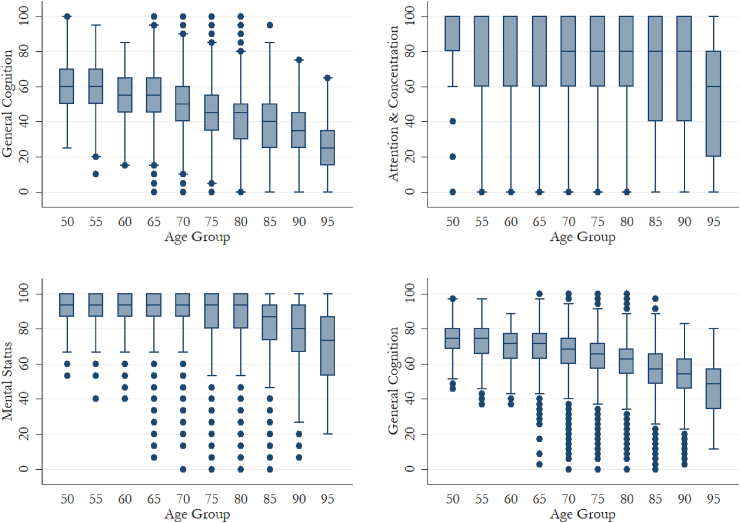
Box plots of the cognitive abilities over age groups (with outliers).

The rescaled cognitive scores represent the comparable percentage of correctly completed tasks. MS tasks were relatively easier compared to EM and A&C tasks. Older adults on average completed 80% and 70% of the A&C and MS tests respectively, while EM has only a mean score around 44, dragging GC towards 60 (Table 1). The mean trajectory in cognitive change over age is presented in Fig. 2.

**Table 1:** Summary statistics for all variables in the analysis: HRS 1998 To 2012 (N = 34,184).

Variables	Mean (SD) or Percentage
*Outcomes: Cognitive Functions (rescaled)*	
Episodic Memory (EM)	48.40 (16.67)
Attention and Concentration (A&C)	75.92 (29.61)
Mental Status (MS)	88.24 (13.34)
General Cognition (GC)	65.47 (12.69)
*Exposure:*	
Education PGS (Unstandardized)	−0.23 (0.14)
Age	74.59 (6.99)
Gender (female)	57.56%
Years of Education (Unstandardized)	12.96 (2.52)
Social engagement	
Low	85.52%
Moderate	12.47%
High	2.01%
Current Smoker	8.31%
Drinking	
Non-Drinker	64.28%
Moderate-Drinker	34.85%
Heavy-Drinker	0.84%
Chronic Conditions	
No Condition	33.48%
1-2 Conditions	57.74%
More than 3 Conditions	8.78%

Higher genetic predictor for education is associated with better cognitive performance, independent of education.

**Fig. 3:**
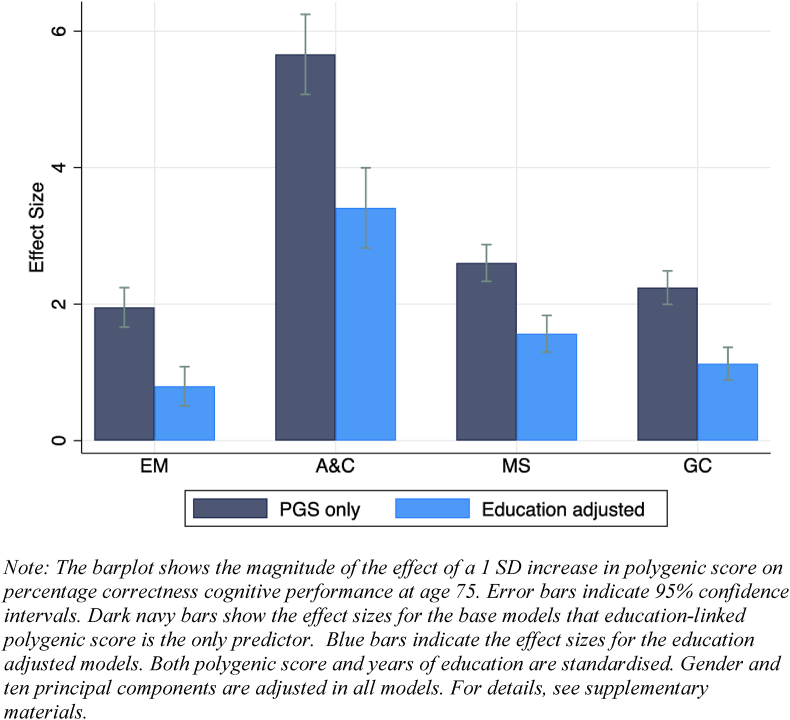
Association between education-linked polygenic score and level of cognitive abilities (n = 5,859, N = 34,184).

Fig. 3 depicts the genetic effect sizes at age 75 from the growth curve models on each cognitive measure. For each outcome, we explore two models: a model with education PGS as the only predictor, and one with education PGS with education adjusted. Age, gender and the first ten principal components are included in all the models.

There is a clear pattern showing that education PGS are independently positively correlated with cognitive levels (Hypothesis 1). HRS respondents with a higher education PGS higher than their peers in cognitive tasks across all measures at age 75. The effect size of one standard deviation increase in education PGS on cognitive ability ranges from 1.9 to 5.7. Estimates are statistically significant (p < 0.001). Since education PGS and educational attainment are correlated (β = 0.31, p < 0.001), unsurprisingly the effect sizes drop after education is controlled for, yet the effect sizes remain highly significant. After taking years of education into account, the effect size of education PGS on EM, A&C, MS and GC declines by 60%, 40%, 40% and 50%, respectively. These results indicate that education PGS influence cognitive performance both independently, and through an education-mediated pathway.

### 3.1 The effect of genetics on cognitive decline varies over age and by domains

**Fig. 4:**
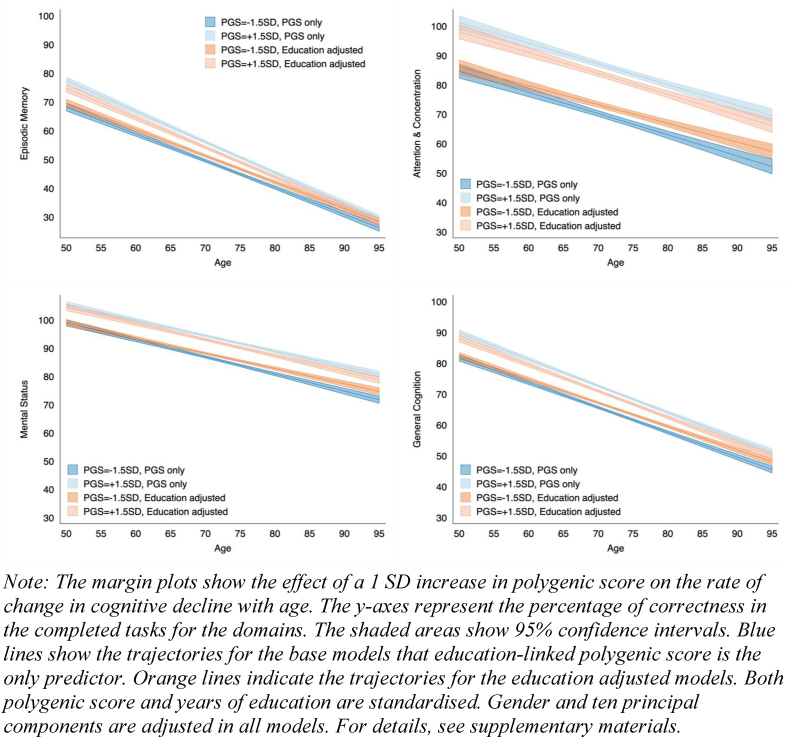
Association between education-linked polygenic score and the rate of change in cognitive decline (n = 5,859, N = 34,184).

The genetic influence on rate of decline is modelled by intercepts and slopes of the growth curve as functions of education PGS and covariates. Fig. 4 displays the predicted age-specific cognitive scores based on the fixed effects of education PGS (with and without controlling for education). Education PGS is negatively associated with EM, and therefore a faster rate of decline (β = −0.04, p < 0.01). The effects indicate that higher education PGS would lead to a faster rate of EM decline in old age. Individuals with higher education PGS scores higher on GC and EM at the late stage of middle age, but the genetic effect diminishes with age. This result contradicts our hypothesis 3, in that the advantage of a higher education PGS on GC and EM fades at old ages. For crystallised intelligence, in line with hypothesis 2, higher education PGS does not change the rate of cognitive decline. Again, after controlling for education, the association between education PGS and the rate of cognitive decline weakens.

For GC, we found that in the education-unadjusted model, education PGS does not have a significant effect on GC decline. Surprisingly, when both education PGS and educational attainment are included in the model, the effect of education PGS becomes stronger and significant at the 0.01 level. Education PGS is associated with a faster GC decline driven by EM. The GC results indicate a suppression effect between education PGS and educational attainment that statistical removal of the education PGS effect could increase the magnitude of the relationship between years of education and cognitive decline.

**Fig. 5:**
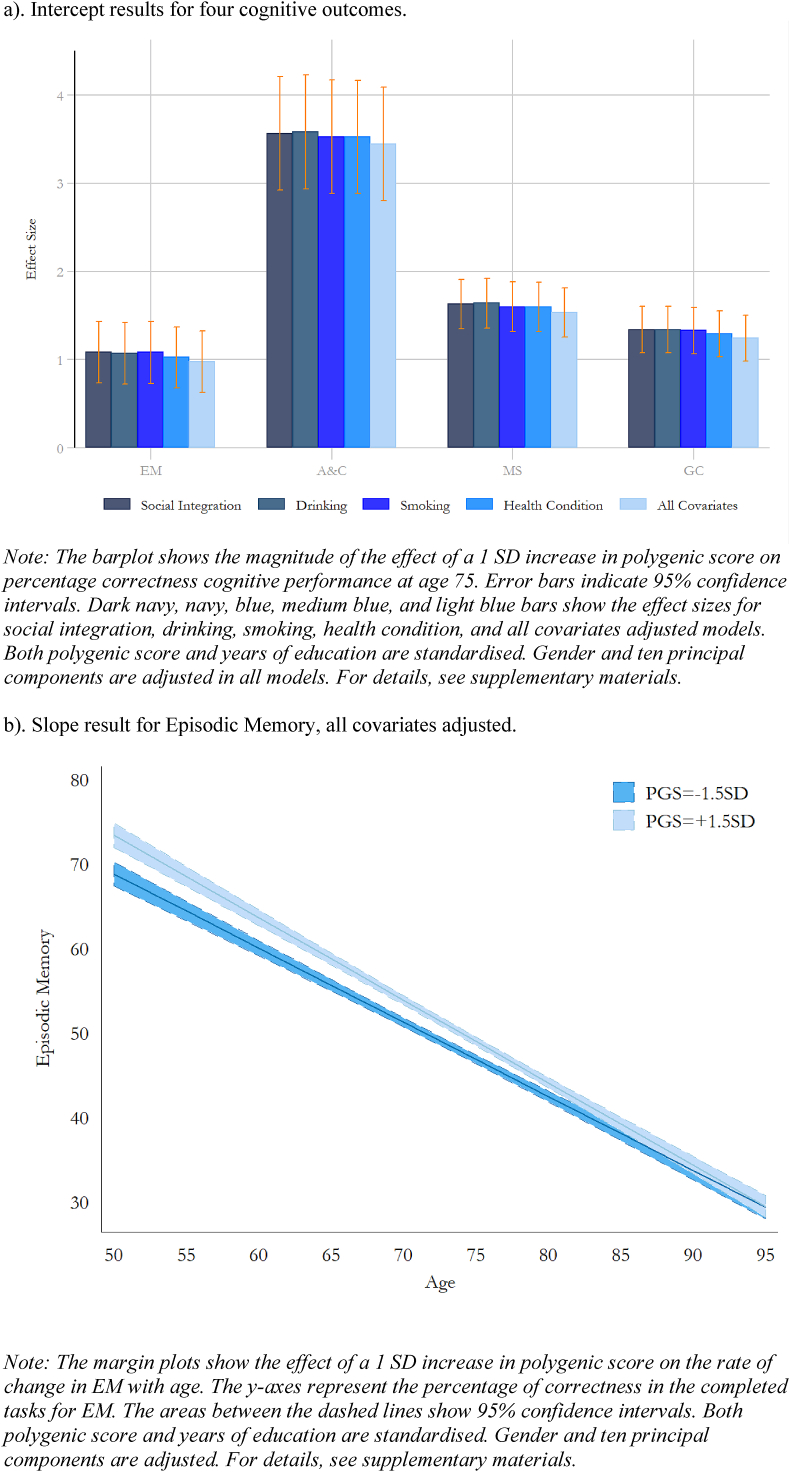
Intercept and slope results from the growth curve models on cognitive outcomes, controlling for covariates (n = 5,859, N = 34,184).

We further examined whether the effect of education PGS could be mediated or confounded by other covariates. We add social engagement, drinking, smoking and health conditions individually to the education-adjusted models. A final full model includes all the covariates. Intercept results for education PGS and years of education are presented in Fig. 5a. For intercept, the effects of education PGS on cognitive performance does not change after adjusting for covariates across all measured cognitive sub-domains. For slope, only education PGS robustly predicts a faster rate of EM decline (Fig. 5b). For general cognition, we found that the effect of EA3 becomes insignificant on the rate of decline after including smoking and pre-existing health conditions.

### 3.2 Sensitivity analyses

We conducted sensitivity analyses to evaluate the consistency of findings. Details are presented in the supplementary materials. First, to examine whether our results are driven by mortality selection, we compared our main analyses with models unadjusted for inverse probability weights. The results from unweighted and weighted models are very similar. Weighted models improve the model fit measured by AIC and BIC. Further, we estimate our models in four birth cohorts (before 1917, 1917–1926, 1927–1936, after 1937). However, the association between education PGS and rate of change in EM loses its significance in every cohort, but the sign remains negative. This may indicate a lack of power from the small sample for each cohort, as the sample size ranges from 859 to 2485. Therefore, even though weighted results reassure us that selection did not produce much bias, we cannot completely rule out the competing explanation.

Second, the nature of the survey-based assessments may produce measurement error in cognitive domains. We plot the coefficient of variation (standard deviation/mean) as an indicator of measurement error (see Supplementary Material). It shows that the coefficient of variation increases with age slightly and becomes fairly unstable after age 90. We excluded the respondents age 90 and above and ran our models again, and our conclusion holds after removing the oldest old. In addition, since cognitive measures are the dependent variables, any measurement error is not likely to bias the estimated effect of education PGS but to reduce the power of the statistical model. Our findings of lack of association hence should be interpreted with caution.

Third, recent studies find that people with higher education PGS are more likely to be born in socially advantaged families (Belsky et al., 2016, 2018; Domingue et al., 2015). Our results are robust after controlling for parental education as a measure of family origin.

Fourth, we control for the general cognition related polygenic score based on Davies et al. (2015). We examine whether education PGS influence cognitive performance via cognition-related genetic mechanisms. The magnitudes of estimates are slightly reduced, suggesting that education PGS predict cognitive performance and decline independently of cognition-linked genetics. The effect of education PGS on each cognitive domain holds even after controlling for covariates, suggesting that genetic effects are not completely mediated by educational attainment and other mediators.

Finally, Keller (2014) has expressed scepticism on the positive findings from gene-environment interaction studies in that potential confounders are not properly accounted for in the statistical models used to test G × E effects. Including the potential confounders as covariates alone in the models may not be sufficient, as this practice does not control for the effects these variables might have on the gene-environment interaction. To show that the results in this study are robust after properly controlling for confounders, we re-ran the G × E models adding the covariate-by-environment (C × E) and the covariate-by-gene (C × G) interaction terms. The results are similar to the main analyses (see supplementary materials).

## 4 Discussion

In this study, we aim to explain the interpersonal variability in age-related cognitive decline with education PGS. Existing research predominantly focuses on quantifying genetic and environmental components of variance in cross-sectional cognitive data and has provided evidence of genetic influences on cognitive ability (Davies et al., 2016). Yet, few researchers have examined longitudinal cognitive change and genetic predictor. Genes are inherited pre-birth and remain the same over a lifespan, but genetic effects on phenotypes can vary over age as a function of gene expression associated with developmental timing or environmental circumstances (Lee et al., 2016). Research to date has not offered information on changes in the genetic contribution to individual heterogeneity in cognitive performance in older age.

Our main research question is whether education PGS is associated with higher initial level and variation in cognitive abilities at the early stages of older adulthood. We analysed data on the trajectory of cognitive performance across three individual and one aggregate domains in over 5000 individuals interviewed longitudinally as part of the HRS. In line with previous literature, we find that education PGS predict a higher initial level of cognitive performances independent of observed years of education, parental education, cognition-related PGS, and other social factors. Our results on the cognitive decline are unlikely to be driven by selection bias. In terms of the rate of cognitive change, the effect of education PGS on episodic memory diminishes over age. We observe no association between education PGS and the rate of change in the attention & concentration and mental status.

Results across a range of cognitive domains suggest that the education PGS is related to significantly higher cognitive functions. Even after controlling for observed years of education, the relationship between education-associated genetic variants and cognitive ability persists. The magnitude of the genetic effect size decreases in education adjusted models. These results are consistent with the evidence from Aysu Okbay et al. (2016), Rietveld et al. (2013), and Rietveld et al. (2014), which suggests that there is an education PGS influence on cognitive ability via both biological pleiotropy and mediated pleiotropy. The genetic variants are associated with a particular neurotransmitter pathway involved in synaptic plasticity, which is the main cellular mechanism for learning and memory (Rietveld et al., 2014).

The analyses of cognitive trajectories caused by normal ageing showed that education PGS is related to the rate of cognitive decline, but the effect is only on episodic memory – a type of fluid intelligence – and driving the same effect on global cognition. Performances in global cognition and episodic memory are better in groups with higher education PGS for those under 85 years old; this difference is completely attenuated over the age of 90 due to faster cognitive decline in the high education PGS group. The findings on cognitive decline are in agreement with recent studies showing that genetic effects vary in cognition with age (Lee et al., 2016). However, the results contradict recent candidate gene analyses, which supports the resource-modulation hypothesis (Laukka et al., 2013; Li et al., 2013; Papenberg et al., 2014). Candidate genes research focusing on a small amount of genetic variants may find a magnifying effect during the ageing process via very specific biological channels (for example, APOE influences memory through low-density lipopropotion cholesterol, high-density lipopropotion cholesterol, and tryglycerides) (Taylor et al., 2011). Such an effect is age-specific. Taylor et al. (2011) report a lack of association between APOE and cognitive function in children. Belsky et al. (2016) adopt a polygenic score approach using growth curve modelling and finds that children with higher polygenic scores performed better on cognitive tests and exhibited a faster pace of cognitive development during childhood. Their result, along with our findings, may suggest that education PGS are more important during younger age, helping individuals to achieve higher education levels, but the protective effect diminishes on episodic memory during the ageing process. Note that our analyses only examine episodic memory as fluid intelligence due to data availability. Future research needs to test more cognitive functions in order to generalise results to other types of fluid intelligence. Future research should also test cognitive change across a longer life span that covers childhood, younger and middle adulthood to comprehensively infer the heterogeneity of genetic influence on the cognitive trajectory.

For global cognition, when we model education PGS and educational attainment separately, both education PGS and education do not have any effect on the rate of cognitive decline. When education PGS and years of education are jointly included in the model, education PGS and years of education both become statistically significant with opposite but more substantial magnitudes of effects. This finding indicates that the education PGS and phenotype confound each other via a suppression effect. Failure to take genetic predictor into account may underestimate the protective effect from years of education, and the adverse effect of genes for education.

Our study suffers from three main limitations. First, the variability in genetic effect may be due to ceiling and floor effects inherent in cognitive measures that narrow the potential range of decline. Mental status as a crystallised intelligence tends to start declining at a later age compared to fluid intelligence and is most pronounced in older adults with pathological brain damages (Albert, 1995). The finding that older adults with lower level of genetic predictor to educational attainment experience a more rapid cognitive decline (compared to a more gradual decline for those with higher education PGS) could be due to ceiling effects in the measurement that limit the variability of change for well-educated older adults with higher initial scores. People with higher education PGS thus enjoy higher cognition for their entire adult life. More sensitive measures that cover greater variability in cognitive function might provide more accurate estimates in future research. Sensitivity analyses excluding the individuals who score the lowest 5% in each measure retained similar results, suggesting floor effects do not compromise the analysis.

Second, although the polygenic score approach is superior to the traditional candidate genes approach in many ways as mentioned above, it is not without limitation. The polygenic socre is based on mostly homogeneous groups of non-Hispanic Caucasian older adults in the U.S. Our findings may not extend to individuals of other ethnic or cultural backgrounds, or later-born cohorts. Furthermore, the education PGS we use explains only a small proportion of Lee et al.’s (2018) estimated genetic influence on educational attainment (Supplementary Material). The genetic discoveries on education PGS do not account for gene-gene interactions or gene-environment interactions. This may lead to measurement error in the score. Our estimates may be thus biased toward zero (Conley, 2016), which provides a potential explanation for the lack of association between education PGS and the rate of change in crystallised intelligence.

Despite its limitations, this study provides an essential contribution to existing knowledge on the variability of cognitive decline by genetics. Our results are consistent with recent research showing that education and cognitive ability are genetically correlated (Belsky et al., 2016, 2018; Wedow et al., 2018). We provide evidence that the causal link between educational attainment and cognitive abilities is subject to genetic confounding. Genetic effects on cognition are not fully mediated by education and independent genetic influences may exist in the relationship between education and cognitive decline. The associations between a genetic predictor to educational attainment and cognitive decline that have been identified are especially relevant because they help to clarify the contributions of observed education and genes to cognitive ageing. Future research should also consider genetic effects when investigating non-genetic factors in cognitive decline. Controlling for genetic effects can avoid omitted variable bias when estimating environmental factors. The finding that the genetic effect on cognitive decline for episodic memory decreases with age represents a need to understand the mechanisms between genetic endowment of educational attainment and cognitive decline from a biological angle.
