# Supplementary material for: The relationship between cognitive decline and a genetic predictor of educational attainment
Source: Soc Sci Med. 2019 Oct;239:112549. doi: 10.1016/j.socscimed.2019.112549 (PMC6873779; doi:10.1016/j.socscimed.2019.112549)
Supplement: Multimedia component 1 [file mmc1.docx]

Supplemental Materials

Table S1. Linear regression models of EA3 on years of education and cognitive outcomes, no controls.

| **Variable** | **Years of Education** | **GC Wave 1** | **EM Wave 1** | **A&C Wave 1** | **MS Wave 1** |
| --- | --- | --- | --- | --- | --- |
| EA3 | 0.31^***^(0.12) | 1.87^***^(0.20) | 1.52^***^(0.22) | 5.06^***^(0.36) | 2.10^***^(0.19) |
| Observations | 5,871 | 3,119 | 5,448 | 5,448 | 3,119 |
| Adjusted R^2^ | 0.095 | 0.027 | 0.008 | 0.034 | 0.036 |

Standard errors in parentheses, EA3 and years of education are standardised.

*** p<0.001, ** p<0.01, * p<0.05

Figure S2. Coefficient of variation for cognitive abilities across age.


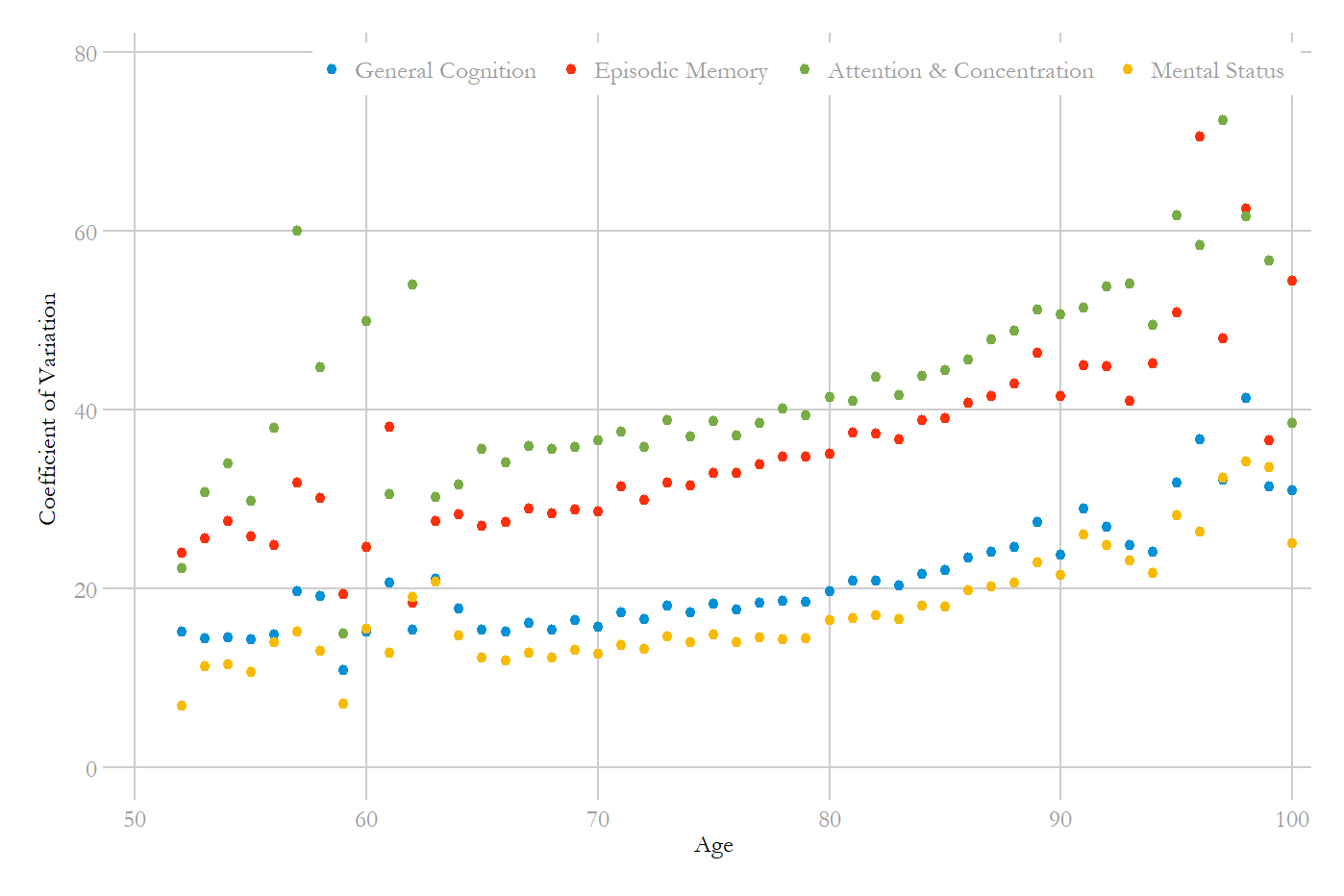


Table S2. Mixed level linear regression on global cognition.

|  | PGS only | Education Adjusted | Unweighted | Below 90 | Family Origin | Cognition PGS | Other Covariates | Environment by Covariate |
| --- | --- | --- | --- | --- | --- | --- | --- | --- |
| Intercept | 63.394^***^ | 63.082^***^ | 63.110^***^ | 63.104^***^ | 63.121^***^ | 63.585^***^ | 63.236^***^ | 62.667^***^ |
|  | (0.186) | (0.171) | (0.179) | (0.179) | (0.179) | (0.223) | (0.182) | (0.415) |
| Age | -0.757^***^ | -0.738^***^ | -0.745^***^ | -0.729^***^ | -0.745^***^ | -0.617^***^ | -0.745^***^ | -0.877^***^ |
|  | (0.019) | (0.018) | (0.020) | (0.020) | (0.020) | (0.026) | (0.020) | (0.046) |
| PGS | 2.234^***^ | 1.115^***^ | 1.127^***^ | 1.121^***^ | 1.049^***^ | 1.050^***^ | 1.132^***^ | 1.086^***^ |
|  | (0.125) | (0.119) | (0.122) | (0.122) | (0.124) | (0.121) | (0.175) | (0.128) |
| PGS × Age | -0.017 | -0.026^*^ | -0.029^*^ | -0.027^*^ | -0.030^*^ | -0.027^*^ | -0.030^*^ | -0.030^*^ |
|  | (0.013) | (0.013) | (0.013) | (0.013) | (0.014) | (0.013) | (0.013) | (0.014) |
| Years of Education |  | 3.589^***^ | 3.577^***^ | 3.587^***^ | 3.584^***^ | 3.422^***^ | 3.584^***^ | 3.350^***^ |
|  |  | (0.125) | (0.123) | (0.123) | (0.123) | (0.123) | (0.123) | (0.139) |
| Education×Age |  | 0.032^*^ | 0.035^**^ | 0.030^*^ | 0.035^**^ | 0.014 | 0.036^**^ | 0.022 |
|  |  | (0.014) | (0.013) | (0.014) | (0.014) | (0.014) | (0.013) | (0.015) |
| Female | X | X | X | X | X | X | X | X |
| Father’s Education |  |  |  |  | X |  |  |  |
| PGS_conition_ |  |  |  |  |  | X |  |  |
| Social Integration |  |  |  |  |  |  | X | X |
| Health Behaviors |  |  |  |  |  |  | X | X |
| Chronic Disease |  |  |  |  |  |  | X | X |
| Covariates by Gene |  |  |  |  |  |  |  | X |
| Covaraites by Age |  |  |  |  |  |  |  | X |
| Var(Chaneg) | 0.32 | 0.31 | 0.34 | 0.34 | 0.34 | 0.32 | 0.34 | 0.33 |
| Var(Initial) | 68.69 | 56.79 | 57.65 | 57.84 | 57.50 | 56.07 | 57.50 | 56.31 |
| Covariance | 1.46 | 1.33 | 1.39 | 1.54 | 1.39 | 1.44 | 1.39 | 1.45 |
| Var(Residual) | 61.93 | 62.24 | 61.81 | 60.77 | 61.77 | 61.62 | 61.81 | 61.26 |
| AIC | 245212.7 | 244384.3 | 251139.4 | 245941.3 | 250405.2 | 250813.3 | 251132.2 | 226174.6 |
| BIC | 245381.5 | 244569.9 | 251325.1 | 246126.6 | 250607.7 | 251117.1 | 251334.7 | 226374.7 |
| Number.Individuals | 5859 | 5859 | 5859 | 5857 | 5842 | 5859 | 5859 | 5859 |
| *N* | 34184 | 34184 | 34184 | 33538 | 34086 | 34184 | 34184 | 34184 |

*Standard errors in parentheses ^~^ p < 0.10, ^*^ p < 0.05, ^**^ p < 0.01, ^***^ p < 0.001*

*Years of education, EA3 score are standardized. 10 principal components are adjusted*

Table S3. Mixed level linear regression on episodic memory.

|  | PGS only | Education Adjusted | IWP | Below 90 | Family Origin | Cognition PGS | Other Covariates | Environment by Covariate |
| --- | --- | --- | --- | --- | --- | --- | --- | --- |
| Intercept | 44.264^***^ | 43.931^***^ | 43.944^***^ | 43.964^***^ | 43.278^***^ | 43.955^***^ | 44.450^***^ | 43.918^***^ |
|  | (0.220) | (0.206) | (0.215) | (0.216) | (0.505) | (0.215) | (0.282) | (0.220) |
| Age | -0.928^***^ | -0.905^***^ | -0.911^***^ | -0.899^***^ | -1.019^***^ | -0.910^***^ | -0.789^***^ | -0.911^***^ |
|  | (0.023) | (0.023) | (0.024) | (0.024) | (0.056) | (0.024) | (0.033) | (0.024) |
| PGS | 1.944^***^ | 0.785^***^ | 0.796^***^ | 0.794^***^ | 0.759^***^ | 0.695^***^ | 0.704^***^ | 0.944^***^ |
|  | (0.146) | (0.143) | (0.147) | (0.147) | (0.155) | (0.149) | (0.146) | (0.215) |
| PGS × Age | -0.045^**^ | -0.037^*^ | -0.037^*^ | -0.033^*^ | -0.032^*^ | -0.039^*^ | -0.033^*^ | -0.036^*^ |
|  | (0.015) | (0.016) | (0.016) | (0.016) | (0.016) | (0.016) | (0.016) | (0.016) |
| Years of Education |  | 3.728^***^ | 3.723^***^ | 3.736^***^ | 3.562^***^ | 3.739^***^ | 3.547^***^ | 3.718^***^ |
|  |  | (0.143) | (0.148) | (0.149) | (0.169) | (0.148) | (0.148) | (0.148) |
| Education×Age |  | -0.020 | -0.018 | -0.020 | -0.030 | -0.018 | -0.034^*^ | -0.019 |
|  |  | (0.016) | (0.016) | (0.017) | (0.018) | (0.016) | (0.016) | (0.016) |
| Female | X | X | X | X | X | X | X | X |
| Father’s Education |  |  |  |  | X |  |  |  |
| PGS_conition_ |  |  |  |  |  | X |  |  |
| Social Integration |  |  |  |  |  |  | X | X |
| Health Behaviors |  |  |  |  |  |  | X | X |
| Chronic Disease |  |  |  |  |  |  | X | X |
| Covariates by Gene |  |  |  |  |  |  |  | X |
| Covaraites by Age |  |  |  |  |  |  |  | X |
| Var(Chaneg) | 0.27 | 0.26 | 0.30 | 0.33 | 0.30 | 0.30 | 0.29 | 0.30 |
| Var(Initial) | 90.21 | 77.78 | 78.89 | 78.97 | 78.79 | 78.59 | 76.97 | 78.86 |
| Covariance | 0.11 | 0.18 | 0.26 | 0.43 | 0.33 | 0.26 | 0.30 | 0.26 |
| Var(Residual) | 131.14 | 131.34 | 130.55 | 130.26 | 129.70 | 130.51 | 130.33 | 130.55 |
| AIC | 267008.7 | 266389.3 | 273763.0 | 268653.2 | 246779.2 | 272961.2 | 273602.0 | 273765.9 |
| BIC | 267177.5 | 266575.0 | 273948.7 | 268838.4 | 246979.3 | 273163.7 | 273905.8 | 273968.4 |
| Number.Individuals | 5859 | 5859 | 5859 | 5857 | 5288 | 5842 | 5859 | 5859 |
| *N* | 34184 | 34184 | 34184 | 33538 | 30836 | 34086 | 34184 | 34184 |

*Standard errors in parentheses ^~^ p < 0.10, ^*^ p < 0.05, ^**^ p < 0.01, ^***^ p < 0.001*

*Years of education, EA3 score are standardized. 10 principal components are adjusted*

Table S4. Mixed level linear regression on attention & concentration.

|  | PGS only | Education Adjusted | IWP | Below 90 | Family Origin | Cognition PGS | Other Covariates | Environment by Covariate |
| --- | --- | --- | --- | --- | --- | --- | --- | --- |
| Intercept | 79.113^***^ | 78.481^***^ | 78.499^***^ | 78.512^***^ | 77.269^***^ | 78.523^***^ | 78.982^***^ | 79.241^***^ |
|  | (0.431) | (0.414) | (0.439) | (0.440) | (1.006) | (0.439) | (0.555) | (0.446) |
| Age | -0.548^***^ | -0.526^***^ | -0.527^***^ | -0.510^***^ | -0.659^***^ | -0.528^***^ | -0.388^***^ | -0.523^***^ |
|  | (0.039) | (0.039) | (0.043) | (0.043) | (0.100) | (0.043) | (0.059) | (0.043) |
| PGS | 5.668^***^ | 3.410^***^ | 3.410^***^ | 3.423^***^ | 3.327^***^ | 3.341^***^ | 3.322^***^ | 2.898^***^ |
|  | (0.299) | (0.296) | (0.300) | (0.301) | (0.308) | (0.305) | (0.300) | (0.432) |
| PGS × Age | 0.013 | -0.017 | -0.024 | -0.011 | -0.025 | -0.024 | -0.017 | -0.034 |
|  | (0.027) | (0.029) | (0.029) | (0.030) | (0.030) | (0.030) | (0.029) | (0.029) |
| Years of Education |  | 7.311^***^ | 7.291^***^ | 7.301^***^ | 6.525^***^ | 7.273^***^ | 7.085^***^ | 7.353^***^ |
|  |  | (0.325) | (0.302) | (0.303) | (0.336) | (0.303) | (0.304) | (0.301) |
| Education×Age |  | 0.098^**^ | 0.102^***^ | 0.096^**^ | 0.092^**^ | 0.104^***^ | 0.074^*^ | 0.110^***^ |
|  |  | (0.030) | (0.029) | (0.030) | (0.033) | (0.029) | (0.030) | (0.029) |
| Female | X | X | X | X | X | X | X | X |
| Father’s Education |  |  |  |  | X |  |  |  |
| PGS_conition_ |  |  |  |  |  | X |  |  |
| Social Integration |  |  |  |  |  |  | X | X |
| Health Behaviors |  |  |  |  |  |  | X | X |
| Chronic Disease |  |  |  |  |  |  | X | X |
| Covariates by Gene |  |  |  |  |  |  |  | X |
| Covaraites by Age |  |  |  |  |  |  |  | X |
| Var(Chaneg) | 0.87 | 0.84 | 0.89 | 0.90 | 0.84 | 0.89 | 0.86 | 0.90 |
| Var(Initial) | 409.96 | 361.68 | 364.03 | 366.05 | 343.35 | 364.10 | 361.72 | 359.34 |
| Covariance | 5.86 | 5.11 | 5.13 | 5.53 | 5.43 | 5.14 | 5.23 | 5.19 |
| Var(Residual) | 399.15 | 399.64 | 397.07 | 394.29 | 388.97 | 397.00 | 396.54 | 396.90 |
| AIC | 305612.0 | 305054.0 | 313361.3 | 307316.6 | 281801.4 | 312471.2 | 313280.2 | 313300.4 |
| BIC | 305780.8 | 305239.7 | 313547.0 | 307501.8 | 282001.4 | 312673.7 | 313584.1 | 313502.9 |
| Number.Individuals | 5859 | 5859 | 5859 | 5857 | 5288 | 5842 | 5859 | 5859 |
| *N* | 0.87 | 0.84 | 0.89 | 0.90 | 0.84 | 0.89 | 0.86 | 0.90 |

*Standard errors in parentheses ^~^ p < 0.10, ^*^ p < 0.05, ^**^ p < 0.01, ^***^ p < 0.001*

*Years of education, EA3 score are standardized. 10 principal components are adjusted*

Table S5. Mixed level linear regression on mental status.

|  | PGS only | Education Adjusted | IWP | Below 90 | Family Origin | Cognition PGS | Other Covariates | Environment by Covariate |
| --- | --- | --- | --- | --- | --- | --- | --- | --- |
| Intercept | 88.821^***^ | 88.548^***^ | 88.512^***^ | 88.529^***^ | 88.228^***^ | 88.555^***^ | 88.974^***^ | 88.886^***^ |
|  | (0.211) | (0.201) | (0.197) | (0.201) | (0.460) | (0.201) | (0.252) | (0.204) |
| Age | -0.513^***^ | -0.503^***^ | -0.495^***^ | -0.484^***^ | -0.660^***^ | -0.504^***^ | -0.378^***^ | -0.501^***^ |
|  | (0.023) | (0.022) | (0.021) | (0.022) | (0.052) | (0.022) | (0.029) | (0.022) |
| PGS | 2.603^***^ | 1.565^***^ | 1.552^***^ | 1.563^***^ | 1.533^***^ | 1.523^***^ | 1.502^***^ | 1.357^***^ |
|  | (0.138) | (0.137) | (0.137) | (0.138) | (0.142) | (0.140) | (0.137) | (0.193) |
| PGS × Age | 0.019 | -0.011 | -0.006 | 0.001 | -0.008 | -0.010 | -0.005 | -0.016 |
|  | (0.015) | (0.015) | (0.015) | (0.015) | (0.016) | (0.015) | (0.015) | (0.015) |
| Years of Education |  | 3.335^***^ | 3.357^***^ | 3.347^***^ | 3.001^***^ | 3.330^***^ | 3.188^***^ | 3.359^***^ |
|  |  | (0.139) | (0.154) | (0.139) | (0.154) | (0.139) | (0.139) | (0.138) |
| Education×Age |  | 0.096^***^ | 0.091^***^ | 0.089^***^ | 0.082^***^ | 0.096^***^ | 0.072^***^ | 0.099^***^ |
|  |  | (0.015) | (0.016) | (0.015) | (0.017) | (0.015) | (0.015) | (0.015) |
| Female | X | X | X | X | X | X | X | X |
| Father’s Education |  |  |  |  | X |  |  |  |
| PGS_conition_ |  |  |  |  |  | X |  |  |
| Social Integration |  |  |  |  |  |  | X | X |
| Health Behaviors |  |  |  |  |  |  | X | X |
| Chronic Disease |  |  |  |  |  |  | X | X |
| Covariates by Gene |  |  |  |  |  |  |  | X |
| Covaraites by Age |  |  |  |  |  |  |  | X |
| Var(Chaneg) | 0.44 | 0.41 | 0.45 | 0.43 | 0.44 | 0.45 | 0.43 | 0.45 |
| Var(Initial) | 82.71 | 72.14 | 72.78 | 73.81 | 68.53 | 72.83 | 72.10 | 71.79 |
| Covariance | 2.71 | 2.40 | 2.44 | 2.66 | 2.49 | 2.44 | 2.55 | 2.44 |
| Var(Residual) | 77.80 | 78.15 | 77.74 | 74.91 | 76.78 | 77.74 | 77.36 | 77.74 |
| AIC | 252396.4 | 251832.3 | 258831.5 | 252862.3 | 232802.1 | 258102.1 | 258499.0 | 258766.3 |
| BIC | 252565.2 | 252018.0 | 259017.1 | 253047.5 | 233002.2 | 258304.6 | 258802.8 | 258968.8 |
| Number.Individuals | 5859 | 5859 | 5859 | 5857 | 5288 | 5842 | 5859 | 5859 |
| *N* | 34184 | 34184 | 34184 | 33538 | 30836 | 34086 | 34184 | 34184 |

*Standard errors in parentheses ^~^ p < 0.10, ^*^ p < 0.05, ^**^ p < 0.01, ^***^ p < 0.001*

*Years of education, EA3 score are standardized. 10 principal components are adjuste*

Rational for inclusion of control variables

Gender differences in cognitive abilities have been observed throughout the population. On average, older men have a better spatial ability than older women, while older women outperform men on reasoning and vocabulary abilities (Denis Gerstorf, Ram, Hoppmann, Willis, & Schaie, 2011). Finkel et al. (2003) demonstrate that women also perform better in episodic memory performance. The rate of decline is similar for both sexes, but after adjusting for the effect of education, women outperform men in processing speed, episodic memory, fluency and knowledge (Gerstorf, Herlitz, & Smith, 2006). This result suggests that women’s lack of access to education earlier in life suppresses their advantage with cognitive tasks. Social engagement is a protective indicator of cognitive ageing (James, Wilson, Barnes, & Bennett, 2011). A recent study from the HRS shows that the social engagement index, taking into account marital status, participation in volunteering work and social interaction is significantly associated with better mental status and self-rated health in later life. However, the same analysis did not find evidence of any impact of the level of social engagement on patterns of cognitive decline over time (Nelson, Noonan, Goldberg, & Buchwald, 2013). Being married or cohabiting with someone and frequently interacting with other people may stimulate cognitive functions and protect the brain from deterioration (van Gelder et al., 2006). The loss of a partner could result in changes in health behaviour such as stress-induced smoking and drinking, and eventually cause adverse health and cognitive outcomes (Vidarsdottir et al., 2014). Krueger et al. (2009) found that a higher level of social interaction in old age is associated with better cognitive performance in general, but this association varies across subdomains. Social engagement has a significant effect on fluid intelligence, but not on crystallised abilities. This body of research underlines the potentially vital importance of social engagement for better cognitive functioning in later years (Ertel, Glymour, & Berkman, 2008). We measure social engagement by an index similar to the one used by Nelson, Noonan, Goldberg, and Buchwald (2013). It consists of measures for marital status, volunteering activities, and contact with family and neighbours. Respondents received one point each for 1) being married; 2) volunteering for religious, educational, health-related, or other charitable organisations at least one hour in the past year; 3) contacting parents weekly or more frequently; 4) contacting offspring weekly or more frequently; and 5) meeting with neighbours weekly or more frequently. The index score ranges from 0 to 5. The sample was categorised into low (scores 0-1), moderate (scores 2-3), and high (scores 4-5) levels of social engagement.

A large body of research on older adults has documented that smoking increases the risk of cognitive decline (Hebert et al., 1993; Ott et al., 2004; Plassman, Newman, Welsh, Helms, & Breitner, 1994). Small or moderate alcohol consumption by older individuals is associated with better cognitive outcomes (Baumgart et al., 2015). Besides, a higher level of genetic predisposition to educational attainment is associated with higher parental socio-economic status (Domingue et al., 2015), which may bring a positive influence on children’s health behaviours and lifestyles. Behavioural risk factors are measured by a binary variable of the respondents’ current smoking status, and alcohol consumption (number of drinks per day). Alcohol consumption is divided into non-drinkers (0 drinks per day), moderate drinkers (1-3 drinks per day), and heavy drinkers (4 or more drinks per day).

Several chronic health conditions have been identified as predictors of adverse cognitive performance, including heart disease, stroke, hypertension, and diabetes. Chronic diseases related to physiological change affecting brain function may explain the association (Qiu & Fratiglioni, 2015). Health risk indicators are measured by determining whether the respondent had ever had heart disease, diabetes, stroke or hypertension. The summarising scores were then divided into three groups: no condition (count = 0), few conditions (count = 1, 2); many conditions (count = 3, 4).
